# Optimising the educational utility of live tissue training in trauma surgery

**DOI:** 10.1186/s12909-025-07846-9

**Published:** 2025-09-10

**Authors:** Cara Swain, Hugo Cohen, Jennifer Routh, Gert Helgesson, Rory Rickard, Klas Karlgren

**Affiliations:** 1 Department of Learning, Informatics, Management & Ethics (LIME) Widerströmska huset, Karolinska Institutet, Stockholm, Sweden; 2 Academic Department of Military Surgery & Trauma, Royal Centre for Defence Medicine (RCDM), Birmingham, UK; 3 School of Veterinary Medicine, University of Surrey, Guildford, UK; 4 Department of Research, Education, Development and Innovation, Södersjukhuset, Stockholm, Sweden; 5 Faculty of Health and Social Sciences, Western Norway University of Applied Sciences, Bergen, Norway

**Keywords:** Simulation, Education, Surgery, Live animal training

## Abstract

**Background:**

Live tissue training (LTT) refers to the use of live anaesthetised animals for the purpose of medical education. It is a type of simulation training that is contentious, and there is an ethical imperative for educators to justify the use of animals. This should include scrutinising educational practices. The authors’ aim was to explore learners’ and educators’ experiences of LTT for surgical learning, employing simulation theory to analyse how LTT can be optimised as a simulated learning event.

**Methods:**

This study used semi-structured interviews to generate data from fifteen physicians who had participated in LTT as learners or educators. The data were thematically analysed using the Framework Method.

**Findings:**

Suggestions on how LTT could be optimised were discussed by the participants across three themes: *Planning*,* Delivering*, and *Experiencing* LTT. The *Planning* theme provides context into what specific learning aims LTT should be used for, and whom this type of learning would benefit. The *Delivering* theme centred around the idea of delivering the curriculum content using various pedagogic methods and framing the animal as a patient rather than a model. The *Experiencing* theme describes the learner’s experience of clinical reality and the importance of real-life survival of the animal during training.

**Conclusion:**

These themes along with simulation theory have been used to create a series of recommendations about how LTT could be optimised to ensure educational quality. Key recommendations include clarifying which learners should attend, restructuring courses to include a brief and debrief, teaching technical and non-technical skills in tandem, and focusing learning on the animal as a patient, in order to promote a realistic experience.

**Supplementary Information:**

The online version contains supplementary material available at 10.1186/s12909-025-07846-9.

## Background

Education of medical professionals that uses live animals as human patient substitutes, so-called “live tissue training” (LTT), is carried out in many countries around the world [1]. This type of education is more frequently a feature of postgraduate surgical training, either as part of a clinical curriculum or a standalone course for learners to attend. Examples of the latter include the international Definitive Surgical Trauma Care (DSTC™) course [2] and the American College of Surgeons’ Advanced Trauma Operative Management (ATOM^®^) course [3].

Simulation involves the use of a simulator in the place of a human patient; in LTT, this is a live anaesthetised animal, which is euthanised on completion of the learning objectives. Research about the effectiveness of simulation is unequivocal – simulation training is associated with improved knowledge, behaviours and particularly skills, across clinical disciplines [4–7]. However, LTT is often considered in a separate category to other educational events using simulator models, likely due to its ethical implications. This type of training is heavily regulated in most countries, often requiring institutional ethical approval, applying the 3Rs of humane animal experimentation – replacement, reduction and refinement [8]. Educators have to justify their decision to use live animals, and should demonstrate consideration of whether such use can be replaced with an alternative, reduced to the minimum required and techniques refined to reduce suffering.

One contemporary argument for the use of LTT relates to realism, with live animals considered to be of higher fidelity for surgical training when compared to other models. It is believed that there is currently no single model that can sufficiently replicate all aspects of a live animal [9], meaning replacement is very challenging. Although there have been significant developments in simulation models for training in laparoscopic, endoscopic and robotic surgeries, there are known and continuing challenges regarding open surgical simulation [10]. One of the major problems in the development of an open surgical simulator is producing a solution that is ‘physics-appropriate’ and can run in real time [10]. However, in a highly-cited systematic review, Issenberg et al. [5] reported the salient features of higher-fidelity simulation that lead to effective learning. Only a single feature (of ten) related directly to the choice of simulator – how the simulator was used was considered more important [5]. 

The majority of educational research within the surgical simulation domain focuses on technical surgical skills [11]. Although not specific to surgery, some qualitative studies have indicated that higher fidelity provided by live animals has educational value beyond the ability to acquire skills, such as management of stress [12, 13], emotional preparedness [14] and increased self-efficacy [12, 15]. 

We conceptualise LTT as a simulated learning event that can be used to train surgeons and surgical teams in multiple areas beyond the acquisition and improvement of technical skills. During LTT, surgery is performed in a multidisciplinary team and participants employ both technical and non-technical skills, and these skills are inter-related [11, 16]. The performance of the surgeon can be enhanced or impeded by their anaesthetic and nursing colleagues also working on the patient, particularly in crisis situations [16]. Failures in non-technical skills, such as surgeons’ situational awareness, have been found to be associated with a higher rate of technical errors [16]. Therefore, it is important that research regarding the educational value of LTT takes a holistic view of surgical practice and explores learning beyond technical surgical skills.

As a training method, LTT is, and will continue to be, ethically contentious. Good educational practice is vital if educators desire to justify their use of LTT. The authors argue that if LTT continues to be conducted, in lieu of a viable alternative open surgical simulator, then there is an ethical imperative that this training is optimised toward the highest educational quality. The aim of this study was to explore learners’ and educators’ experiences of LTT for surgical learning, employing simulation theory to consider how LTT can be optimised as a simulated learning event.

## Methods

### Study design

We conducted a qualitative research study using semi-structured interviews, analysed using the Framework Method [17]. The work was approved by the Swedish Ethical Review Authority (Dnr 2022-05-03).

### Recruitment of participants

Participants were invited to participate if they had prior knowledge and experience of courses involving LTT, either in the role of learner or educator. The lead author was observing LTT simulation courses (*n* = 7) in several centres across five countries for another study [18]. Faculty educators at each observed course were invited to participate by direct email contact by the lead author, or via invitation disseminated on behalf of the research team by the course director. Where permitted (at three of seven courses), learners who attended the observed courses were also invited to participate. This initial phase of recruitment resulted in eight interviews being conducted.

To further recruit participants who had experience of LTT, information about the research study was disseminated by the Royal College of Surgeons of England to personnel involved in teaching on their trauma courses. This second phase of recruitment resulted in nine interviews being conducted. Two of these participants were excluded on completion of the interview as their experience related either to live animals involved in clinical research or the use of deceased animals in surgical trauma simulation. Fifteen interviewed participants were included in the analysis; their characteristics and experience with LTT are specified in Table 1.

**Table 1:** Characteristics of included participants

**Participant characteristics **	**Number of participants ** **(total = 15) **
Profession	
Trauma or emergency surgeon	10
Other specialist surgeon	3
Anaesthetist	1
Emergency medicine physician	1
Seniority	
Consultant	13
Fellow or registrar	2
Gender	
Male	11
Female	4
Country of employment	
UK	7
Sweden	5
USA	2
Multiple countries	1
Main LTT experience	
Learner	9
Educator	6
Number of occasions participated in LTT	
1-2	9
3 or more	6

### Data collection

Interviews took place online between April 2023 and February 2024, using a prepared semi-structured interview guide (supplementary file). The overall topic related to the participants’ perceptions of how simulation involving a live animal was used for learning about trauma surgery. Learner participants were asked about their expectations and motivations for attending training, what they perceived they learned across different domains (i.e., cognitive, psychomotor, affective), and how their learning translated to their clinical practice. Educator participants were asked about their use and involvement with LTT, and for their insights on learners’ experiences. The interviews were conducted in English by the lead author (CS). Audio recordings were transcribed using voice recognition software and transcriptions were manually corrected and independently confirmed by two researchers. The fifteen analysed interviews were 27 to 59 min in duration with a median duration of 37 min.

### Data analysis

Data were analysed in iterative steps following the Framework Method [17]. This is a systematic, iterative and transparent approach to thematically analysing qualitative textual data, which features two components: creating and applying an analytic framework. The framework is created from the data and then applied in order to comprehensively structure and summarise data use, while maintaining the original context. It is especially relevant for studies that aim to inform policy and practice.

There are five stages to the Framework Method: (1) data familiarisation; (2) creating a framework; (3) indexing data using the framework; (4) charting and summarising indexed data into a matrix; and (5) interpretation of the thematic patterns. Although described sequentially, the early stages can be fluid as applying the framework to index data may necessitate revision of the framework, with amendments to the structure or the definitions and boundaries of constituent categories.

To become familiar with the data, CS listened to all interviews and re-read all transcripts; KK, JR and HC each read three different transcripts. CS and HC open-coded all transcripts, with KK and JR providing suggestions for codes and categories based on familiarised transcripts. Codes capture the essence of an identified concept, with categories grouping similar concepts together, resulting in an indexed dataset. Informed by group discussion and simulation pedagogy literature, CS developed an initial analytical framework directed toward the research aim, which was then trialed, reviewed and iteratively refined by the researchers. The final result was a framework formed of 20 categories and 91 codes, which was inputted into the software program NVivo.

All transcripts were re-coded (indexed), with minor amendments to the structure of the framework as directed by the data. Indexed data were charted into a matrix using Microsoft Excel, which summarised, rearranged and ordered participants’ interviews by category. Patterns across the dataset and between framework components were then explored, including the presence of data intensity (congruent ideas across many participants) and variation apparent within sub-categories, by reviewing the matrix. Overarching interpretative themes were generated, guided by the research objective. Illustrative quotations were selected, reflective of each theme’s comprised categories, with minor linguistic corrections made to enhance readability. As differences between learner and educator perspectives was considered relevant, study participants whose experience of LTT was in the role of learner are reported with numbers 1–9, educators are reported as letters A-F.

### Reflexivity

The research team comprised a variety of backgrounds, experiences and perspectives relating to LTT, and medical simulation in general.

Three members of the team, CS, HC and RR, are practicing surgeons so considered the data from their own educational perspectives with a professional understanding of the context and the potential learning outcomes. CS is a doctoral researcher whose research project has specifically focused on the educational use of LTT. In addition to conducting interviews with learners and educators, she has observed courses using live animals in a variety of countries, but has not participated as a learner herself. HC has an academic interest in medical education, with no personal experience of LTT; RR is a professor of surgery with personal experience of LTT who instigated research into its educational use with a view to its considered replacement and refinement. Both consider that the practice has a role in trauma, at least until there are significant advances in simulation technologies.

JR is a veterinary surgeon and lecturer in veterinary education. Her professional background meant she knowingly viewed the data from both an animal welfare perspective and drawing on her own experiences as a clinical educator. With no personal experience of human surgical training, and having taken a professional declaration that she must above all, constantly endeavour to ensure the health and welfare of animals, she approached the analysis with a scepticism of LTT.

KK is an educational researcher whose interests relate to simulation and use of technologies in learning. He has been involved as a researcher with simulated learning across various medical domains, including observing training involving live animals. GH is a professor of medical ethics who has no personal experience of LTT and prior to this research project was unaware of its existence. Both KK and GH, along with CS, hold a neutral perspective regarding LTT considering themselves neither for or against the practice, but agree with the principles of the 3Rs of humane animal research and experimentation.

## Findings

Optimisation of LTT was discussed by the participants across three themes: *Planning*,* Delivering*, and *Experiencing* LTT. These themes have been structured to reflect stages of participation in a simulated learning event, but there are notable similarities within some of the sub-categories. The *Planning* theme provided context into what LTT should be used to learn and whom this type of learning would benefit. The *Delivering* theme centred around the idea of delivering the curriculum content and featured categories relating to logistics and set-up, pedagogic methods and framing of the animal. The *Experiencing* theme describes the learner’s reality when they participate in LTT. Tables 2, 3 and 4 summarise the themes from the framework analysis, with the presence of data intensity indicated in bold. Quotes from the participants have been used throughout to illustrate the findings; limited changes to their words have been made to aid reader understanding, and are indicated by text enclosed in square brackets.

**Table 2:** Planning theme

Categories	Sub-categories & codes	Representative quote
Why do learners decide to attend a course?	Increase clinical experience - **Mitigate lack of exposure in clinical practice** - Practice rare of difficult procedures - Try something for the first time - **Increase comfort and develop confidence**	“It’s better to I guess, have practice outside of the hospital and try and be more comfortable doing certain procedures than having to do it right then there on the spot where you have little to no experience […] One of the rare things that we see is any type of trauma, like major vessels, or even to like the heart, pretty rare that we see that, and so you don’t get as much experience clinically” [L-3]
	Improve patient care - Train without risking human life - Improve patient outcomes	“This is a difficult situation, you put people in, when penetrating trauma happens to a person. And you have no chance to train that beforehand. And it is not fair to either the patient or the doctor, I will say. And this is a way we can train it.” [E-E]
	Personal or professional drivers - Recommendation or requirement - Time away from clinical work	“I did it because somebody said to me, oh, you know, go to the DSTC… and it was cheaper […] and I wanted to travel. So those are really my sort of motivators should I say, as well as actually somebody saying to me, go on it.” [L-7]
What type of learner is the course for?	Defining the surgical learner - **Appropriate level or stage of training** - Past or current experience managing trauma - Knowledge of anatomy and physiology - Technical skills ability	“I think this course is for people who have a specific interest in being trained in resuscitative surgery on a training pathway that’s neared completion or has completed, such that they’ve got a skill set that would then enable them to actually get the most out of this course.” [E-F]
	Multidisciplinary learner involvement - Anaesthesia - Emergency medicine - Peri-operative nursing	“One thing is that I would love to have this this animal lab in cooperation with the anaesthesiologist, to actually give the anaesthesiologist an opportunity to, you know, to treat the deranging physiology… to make the animal cope better with the surgery.” [E-D]
What are the potential learning objectives?	Technical skills - Haemorrhage control - Identify injuries - Practice complex cases - Manage complications - Specific skills (perform a thoracotomy; cardiac injury management; repair major vessel injuries)	“That’s the hardest part I think of surgery when there’s audible bleeding, and you just have to get into it right away and get control. […] The use of a live animal model is actually probably the best way to actually get in touch with good bleeding and how to stop bleeding and how to take care of bleeding.” [E-D]
	Non-technical skills - **Team-working** - Communication - Decision-making - **Stress and pressure management** - Time management - Situational awareness and judgement - Resource management	“You’ve got blood and you’re gonna, you know, have a bit of stress in actually trying to get control of this situation […] of having to now operate under pressure. And something deteriorating in front of you, is, you know, what, the whole point of the course is really. […] I think this also, you know, the human factors, how you deal with the response of it, I don’t think is taught enough or embedded into this type of learning. You know, the emotional response, and then how you talk about it after is important. [E-F]
	**Integrated technical and non-technical skills**	“It’s the technical skills under the pressure of having consequences. That is not replicable in other sort of situations. Because anybody with practice, you can teach a monkey to stitch. It’s just a repetitive motion. The thing that’s the key is, can you do it when shit’s hit the fan.” [L-4]
Which simulator to choose?	Risks and disadvantages of using a live animal - **Premature death of the animal** - Waste of animals - Resuscitative differences - Anaesthetic differences - Anatomical differences - Financial cost - Secrecy - Legislative requirements	“My pig […] it died very quickly, before we got to do very much things. […] I wouldn’t say that when the animal died that made it [LTT] worse. It just didn’t have the benefits that it could have done.” [L-6]
	Alternative options (mannequin; human cadaver, including perfusion; dead animal)	“A cadaver is like an excellent model because you have all the anatomy and, but it’s more expensive, it’s difficult, you have a fresh frozen, which is the one that would like, give you the sense that like it feels like a real human tissue. […] In the cadaver, it’s less engaged on, on like, trying to save this patient, its’ more like trying to find the structure […] they can do the same stitch, and they can take like, five minutes doing it because it’s not bleeding…” [E-A]
	Combination of modalities – different simulators for different learning objectives	“I don’t think any single surgical platform be it anything from, you know, live tissue to deceased tissue to perfused deceased tissue to prosthetic tissue, will cover each of those areas. So you need to try and work out where the Venn diagrams overlap for your particular audience and choose the two models, max of two models, I would say.” [E-B]
Who should be recruited as faculty?	Reasons for faculty selection - Credibility - Role-modelling - Teaching ability - Invite or other reason	“You have a really experienced instructor stood next to you, talking you through and helping with what you do. […] The teachers are basically rock stars. You know, they are dedicated trauma surgeons who do this with a high volume of penetrating trauma… […] You know, you can be credible and not be a good teacher as well. And I think they have a combination of both, you know, they were really nice people, highly experienced in what they do.” [L-8]
	Teaching style and choices - Activity - Ethos - Presence of hierarchy	“I don’t know what how my colleagues run it… there’s also a personal thing you give into each of the tables. […] It’s very different styles. So some people are much more dogmatic like, step one, step two, step three, blah, blah, blah, some others are more like, they want to get involved on that. Yeah, I think that that also might vary a lot. [E-A]
	Benefits of being a faculty member - Enjoyment - Learning from others	“It is something which allows you to learn as well, because you’re always learning from your peers and from junior colleagues who ask basic questions such as ‘why? why are you doing it like that?’ which allows you to challenge your paradigm and to be able to refresh your own understanding of why you do things. [E-B]

Bold entries were contributed to by 10 or more participants

**Table 3:** Delivering theme

Categories	Sub-categories & codes	Representative quote
Logistics and set-up	Ratio - Theory to practice - Learners to animals - Learners to faculty	“We needed to take turns, and we were four people sharing the time… If we would have maybe been two people per animal, we would have been able to do to do longer interventions or longer cases.” [L-1]
	Veterinary care	“Their vet, I can’t remember his name […] a really passionate guy who’s really dedicated to doing this as well as humanly impossible. And so you kind of buy in, you buy in a little bit more to […] this is important that we do this properly.” [L-9]
	Duration of LTT	“I always tend to feel that the courses are short, or too short for… I would like to spend more time within the lab.” [E-A]
	Procedures trained (type, order, adaptation)	“It’s also the case that you plan your skills according to time. So you do the most advanced at the end, of course.” [E-E]
Pedagogic methods	Peer learning	“I think for, for myself, for technical skills in terms of what I wanted to accomplish, I think it’d be better to be someone at my level, or even working with someone above my level, so I can gain more information from them.” [L-3]
	Interactive discussion with experts	“The discussions one gets out of seeing you know, three or four trauma experts talking about their indications for you know, restoring bowel continuity versus delaying etc following a long injury are really illuminating for junior learners to see.” [E-B]
	Demonstration and observation	“It was more of an observer role, but on the other hand, it was very, very eye-opening for me, because this is not, not every day you see, lateral thoracotomy. […] It was a very good learning experience. And this time, we also had the chance to be with the surgeon in the wound, and him describing the manoeuvres he was doing.” [L-5]
	Reflecting on past experiences	“We could talk about how it related to our hospital and sort of, we could think about patients that we’d all worked on together and how it would have made a difference for them.” [L-6]
	Practical and sensory experience	“While you’re doing it, you know, you’re seeing how the heart beats, you’re seeing how it feels, how it bleeds, how it’s reacting on the monitor. Those are all things that you know, you take into account and learn from, and then can apply it while you’re in the hospital to patients.” [L-3]
	Repetitive practice	“It’s about just making sure you get the steps and the foundations right. And then you just replicate, replicate, replicate.” [L-4]
	Formal assessment	“I think the post test was not very useful. You know, the pretest, I don’t think is very useful because that was more so of, cognitive, of like, you know, this patient is in this scenario with this vitals and this injury, you know, what do you do next? […] It’s a set of skills, and you come out with more skills than when you first went in, and whether or not you became better at those skills, I think is really more of an important test than just a written cognitive test.” [L-3]
Framing of the animal	Animal as a (model) resource	“We did use two pigs during the day anyways. And I think our first pig was perfectly fine. I think we could have kept it. So now we didn’t because it was just decided beforehand.” [L-2]
	**Animal as a patient**	“I never lost an animal before this… because it’s a pride for every teacher. […] You know, we give them names. We give them names. They are told to tell them their names. We never talk about pigs. Never.” [E-E]
	Utilitarian ethical view of animal use*	*The material in these codes was considered so rich that a sub-analysis was performed exploring the ethical views of LTT users and this has been reported as a sub-study.

Bold entries were contributed to by 10 or more participants

**Table 4:** Experiencing theme

Categories	Sub-categories	Representative quote
Clinical realism	**Bleeding, perfusion and tissue tactility**	“Having the experience of doing it with the actual, with those, with the bleeding and the movement of the heart, which is just so different from a cadaver, but much more realistic to real life.” [L11]
	Reactive physiology	“I think it goes beyond just the bleeding. [...] I think just having the active bleeding is... and the fact that you will see the blood pressure, the heart rate change and sort of motivate you to do something.” [L12]
	Provision of physiological parameters	
	Variable and unpredictable nature	“A couple of people's pigs didn't last the morning sessions. Ours was lucky for the first day, I'm not, I still don't really know, how it managed to survive what it did for the time it did.” [L9]
	Actions have consequences	“If you do the same, you know, for example, trying to dissect out the femoral artery and you inadvertently, you know, nick the femoral vein, you're going to know about that... in a cadaveric model, you know, okay, ooh, that was the vein, you go, shouldn't have cut the vein. [...] You need to have some consequence of dealing with a blood vessel and, you know, an IVC in a pig is going to bleed a lot, in the same way as an IVC in a human's going to bleed a lot.” [E17]
Contextual realism	**Working as a team**	“To cooperate with other doctors at different levels, whether we know each other or not, it feels like our normal job. And we, I would definitely say that the parts when we didn't know what the what the problem was, and then we were to try to find it and solve it, it felt realistic.” [L6]
	Correct surgical equipment	“She was very competent, and all that. But they, they didn't have the right stuff. So it was a little bit hard for her.” [L4]
	Complexity and problem-solving	“We didn't know what we were expecting, what kind of damage we were expecting, which was better than we'd have to, you know, search for it. Which is more realistic.” [L6]
	Accuracy of the scenario	“The scenario was explosion and gunshot wound, and patient being dropped off from Warzone to, like, remote area. So we were down in the basement doing like, triage and haemorrhage control. And then we moved up in the laboratory and into the OR room and had different simulation during the day there. [...] It was like realistic trauma, like war scenario training for me.” [L10]
	**Emotional engagement**	“It's not the same, but it is the most realistic experience I've certainly had, of the pressure implications and potential stress that's a live operation on a human when you're doing the operation, can replicate.” [L9]
	Performing as a surgeon	“When it came to the to the animal model, then they really relaxed actually, because that's what surgeons, do they operate...” [E5]
	Competing with others	“That brings out the, sort of the competitive edge of surgeons, nobody wanted to kill the pig, everybody wanted to save the pig. And everybody wanted to do what we were there to do safely.” [L9]
Realism and real-life survival	N/A	“The benefit of them having alive tissue is that okay, you now are actually going to do it. [...] Just how realistic, I mean it sounds ridiculous, but you know, they're obviously a live animal. [...] You've got a vested interest in trying to keep the animal alive for as long as possible.” [E17]

Bold entries were contributed to by 10 or more participants

### Planning

The *Planning* theme was comprised of five categories, interpreted as questions that an educator might consider when designing a simulated learning event. These categories/questions are:

- Why do learners decide to attend a course?
- What type of learner is the course for?
- What are the potential learning objectives?
- Which simulator to choose?
- Who should be recruited as faculty?

Table 2 demonstrates the categories comprising this theme and provides more granular detail in the form of representative quotes from the study participants.

Participants felt that LTT is beneficial for surgical learners to practice technical skills, specifically rare skills associated with haemorrhage control, which they are not otherwise exposed to in clinical practice, and lack confidence in executing. Learners can gain experience and try something for the first time, in a safe environment, without risking human patients’ lives.

*“If it’s the first time you’re going to put a stitch to [the] heart… probably it’s not the best time to do it on a bleeding shocked patient.” [Educator A]*.

*“The goal of simulation is to practice where the stakes are not a human life” [Educator C]*.

Learners attending an LTT course should be at an appropriate stage of training and have sufficient experience in managing trauma, with a reasonable base of technical operative skill. There is an expectation that learners will know human anatomy and physiology and have an awareness of the techniques being practiced. One educator [A] stated LTT was to *“rehearse the technique and clarify doubts”*. It is important that they do not attend until they are ready. There was no clear consensus from participants about when this would be, but typically was felt to be later in surgical training as *“translating the benefits of [the] course… requires a certain level of maturity*,* a certain level of skills” [Learner 4]*.

Training in non-technical skills can be integrated with training in technical skills. Participants considered that performing a thoracotomy, and managing cardiac and major vascular injuries were highly valuable in this regard. LTT is felt to be an excellent learning opportunity to perform as part of a team and operate under pressure. The inclusion of learners from multiple healthcare disciplines such as anaesthetic practitioners, emergency physicians, and peri-operative (‘scrub’) nurses was felt to be beneficial, although not necessarily for their learning, rather as support to the surgeons’ experience.

*“Catastrophic bleeding from a brachiocephalic vein or a ruptured aorta*,* because it’s time-sensitive*,* and you have to do something – you’re not going to know what you’re going to do until you’re in that situation.” [Learner 4]*.

*“I had a course a while ago where a student just turned away and walked off when the heart was bleeding… it kind of shocked me a bit. He came back*,* and it worked. Maybe this is a way of teaching that? How can you learn this if you never see tough bleeding situations in your day-to-day life? You should be able to handle [these] situations in some way at least.” [Educator E]*.

There is inherent risk associated with using a live animal; principally, that the animal can die. The perceived pressure relates to being able to perform the skill, and keep the animal alive. However, there are disadvantages that are recognised such as anatomical dissimilarities, differences in anaesthesia and resuscitative practices, financial cost and legislative requirements, among others. The death of the animal, specifically premature death prior to the completion of the training, was recognised as an issue that can impact the learner’s experience.

*“If you do have inadvertent bleeding or bleeding that you have caused*,* if you aren’t able to resuscitate them*,* as was borne out in one of the two models that were used*,* you know*,* the animal succumbed sooner than we had hoped.” [Educator F]*.

There was an overall awareness that animal use should be replaced with alternatives where possible, especially where the primary focus is on learning to acquire technical skills rather than train all elements of surgical practice. A learner [9] commented that *“you can do an awful lot of training on mannequins before you reach [LTT]”.* Most participants felt that a combination of simulator modalities is beneficial, typically use of a cadaver for revision of surgical technique with human anatomy, followed by live animals for practice of haemorrhage control in a team setting.

Choice of faculty is important within the planning process, with one learner [8] commenting *“there’s no such thing as a bad student*,* there’s only a bad teacher. You can have courses where you don’t really learn anything and that’s down to the teacher and not to you really.”* Many of the faculty educators volunteer out of enjoyment and to learn from others, with one educator [B] also stating that there is *“a duty or a responsibility to train the next generation.”* The learners value educators who are competent and credible trauma surgeons, but their approach should be that of a mentor with supportive peer interaction, and they should be able to adapt their style to suit the learner whom they are training.

### Delivering

The *Delivering* theme features three sub-categories: logistics and set-up, pedagogic methods, and framing of the animal (Table 3). The majority of participants felt that a course involving LTT should have a greater focus on practical application rather than theoretical, more didactic, teaching about trauma surgery. The preference expressed by study participants was for learners and faculty to work as closely to a 1:1 ratio as possible, utilising one animal for as long as possible. This approach would maximise the amount of time available for a surgeon to train and receive focused guidance. The importance of veterinary care was recognised, which both added gravitas to the training and introduced potential learning opportunities through interactions with veterinary practitioners.

When determining how the procedures within a curriculum should be practiced, it was noted that there needs to be a balance to sustain the animal, *“starting on the more minimal procedures and working up so that if there was a high-risk manoeuvre being taken*,* that was later on in the day” [Educator F].* However, this means that the valued, riskier procedures that the learners wish to practice may not be achieved. One learner [3] explained that *“repairing the IVC*,* or even some sort of aortic injury… different techniques of repairing it*,* was definitely one of my goals that we didn’t accomplish and I think a lot of it had to do with time constraint.”*

There were multiple pedagogic methods reportedly used during LTT, but it was the practical and sensory experience along with interactive discussion, the opportunity to ask questions and receive feedback in the moment, that was perceived by the respondents as most positive and desirable. Nonetheless, learners and educators also reported benefit in observing faculty and their fellow learners on the course and reflecting on their own ability and experiences.

*“I think*,* for example*,* packing of the liver… I mean*,* you can read in a book… but you got very hands-on advice. You hold like this with your left hand and you take this with your right hand*,* and you can do [it] like this… I think if you see and you practice and you hear something*,* it really stays in your memory.” [Learner 2]*.

*“I think if the learners were really paying attention*,* everybody at the table should have been actively learning through observation*,* right? So it’s not just about me doing a nephrectomy*,* it’s about everybody on the team… learning*,* absorbing. Hmm*,* he or she did that*,* I wouldn’t have done that. Why did they do it that way? Is that the best way to do it?” [Educator C]*.

The animal can be framed as a model (a resource) or as a patient, alongside a utilitarian view that although this type of training has ethical implications, it is required in order to train to save human lives. Educators tended to explicitly refer to the animals as models. Few participants referred to animals as patients directly, but it was recognised that *“we are using animals that have feelings and thoughts”* [Learner 2] and that *“everyone wants to keep their animal alive”* [Learner 3]. One learner [2] stated *“it’s what we do*,* we try to keep patients alive*,* we want it to bleed as little as possible”* indicating that in their experience, they considered the pig akin to one of their patients. By considering the pig as a patient throughout, the curriculum is delivered in a manner that is less akin to simulation training and more like real-life surgical practice, and impacts on the learner’s experience of LTT (third theme).

*“It’s just the fact that something is alive. It has a pulse*,* you can see it’s living*,* even though it’s not a human being but there is something I think innate within us*,* if not all of us*,* but most of us, that you know you do have this sort of bit of a consideration of the fact that something is alive and you have to treat it well because it is still a luxury for an animal to be available for us to be able to use to learn… You felt more*,* you know*,* there was a sense of what you’re doing really matters.” [Learner 7]*.

*“I learned how to fix an IVC bleed in an animal model. And then the first time I had to do it*,* for real*,* and it worked… still leaves*,* you sort of shaking a little bit afterwards. But actually*,* when you reflect on it*,* that wouldn’t have been possible without the sacrifice that [animals] have made… I think there’s a whole thing around the*,* what’s the right phrase*,* the*,* reverence of what we’ve been given*,* and what we have to have done to have got that.” [Learner 8]*.

### Experiencing

The *Experiencing* theme is formed of three sub-categories that describe the learners’ experience of participating in LTT: clinical realism, contextual realism, and real-life survival. These are further described in Table 4.

Aspects of clinical realism that were considered to be valuable contributions to the experience were real-life bleeding, tissue perfusion and responsiveness, and the presence of reactive physiology. Their actions have consequences. One educator [D] was clear: *“a live animal model can bleed out if you screw up. So you have to treat bleeding!”* When the learners performed a manoeuvre to stop bleeding, they would receive instantaneous feedback from the animal itself, which was also indicated by the physiological parameters. It was recognised that animals, like human patients, are unpredictable; this is different to other types of simulation training using a standardised model.

*"It’s quite easy to kill a pig, you know, and so therefore if you make the scenario a little bit too challenging, suddenly your live pig is no longer alive."[Educator B]*

There were aspects of contextual realism that enhance or detract from the learning experience. Team working, in particular realistic interactions between surgeons and anaesthetists, were considered important, along with the clinical accuracy of the scenario. The lack of appropriate surgical equipment was considered problematic, with one educator [D] commenting that “*there’s a shortage of*,* you know*,* specific vascular tools or even decent needle holders… it is surgery with a bit less functional equipment.”*

The emotional engagement with the training was consistently highlighted as a feature that added to the realistic experience. One learner [7] thought that LTT *“just focuses the mind a bit more… it’s one thing operating on a non-live model*,* but if something is alive*,* I feel your empathy*,* your humanity comes in a bit more*,* and you’re much more considerate and careful versus if whatever you’re operating on is not alive.”* An educator [F] observed *“that emotional response to what you’re doing is*,* I think only borne out when you have the live tissue… [LTT] is so intense*,* you know*,* the concentration level is far superior*,* much quieter*,* people are really focused on helping do the operation properly.”*

The participants experience LTT as highly realistic and as a matter of real-life survival. One learner [1] reported being completely absorbed in the training, *“for me*,* the day was like five minutes from the morning till late afternoon… I wasn’t even thinking about what time it was*,* I mean*,* it’s like when you are in the OR!”* One educator [C] explained that *“when it’s an animal*,* there’s no suspending of belief*,* because it is an animal*,* it is animate and it is alive.”* LTT is perceived by many as the closest simulation training possible, which is a highly desirable experience.

*“If you are as close to as it’s going to be in real life*,* then when you start to have to do it for real life*,* it’s not so much of a jump. But if all you’ve done is stitch a cadaver’s blood vessel back together again*,* the moment something’s bleeding torrentially*,* and you have that extra little bit of pressure thrown in*,* it’s not close to what you’re going to have to do.” [Learner 8]*.

Although the findings have been presented as three themes in a sequential order, the boundaries between these themes are not sharply delineated. Sub-categories within them undoubtedly influence the overall experience of participating in an LTT event. For instance, aspects of the *Planning* theme, such as multidisplinary learner involvement, can impact on the contextual realism of working as a team (*Experiencing* theme) and the recruited faculty’s teaching style and choices will influence pedagogic methods (*Delivering* theme). However, it should not be assumed that the influence is unidirectional. Aspects of realism reported within the *Experiencing* theme, especially real-life survival directly relates to the framing of the animal as a patient (*Delivering* theme) and also the risks and disadvantages of using a live animal (*Planning* theme), demonstrating bi-directional interactions between certain sub-categories within the analysis.

## Discussion

This study reports findings from learners and educators involved with simulation training courses that use live animals to learn to manage surgical trauma. These findings have been organised to report three different stages of a simulation-based learning activity: planning, delivering, and experiencing.

Simulation has been demonstrated to be more effective than traditional clinical education practices [19]. Nestel and Gough [20] have described a systematic approach to designing simulation-based learning events that includes six stages: preparing, briefing, simulating, debriefing, reflecting and evaluating. Our *Planning* theme is akin to the preparing stage, with the *Experiencing* theme reflective of the simulating stage. The *Delivering* theme has aspects which could be considered across the three stages of preparing, simulating, and debriefing. Our aim was to explore learners’ and educators’ experiences of LTT for surgical learning to consider how LTT can be optimised as a simulated learning event.

Interestingly, our participants have not reported on aspects of simulation training that are known to be valuable, such as the benefit of debriefing and reflecting. An empirical ethnographic study of surgical learning in LTT conducted by the lead author [18], identified that the predominant emphasis by both educators and learners is on the acquisition and practice of surgical skills. LTT is delivered more akin to a clinical learning experience than a simulation. Feedback is provided in-situ to correct behaviours, with no systematic analysis or formalised debrief after the event and minimal available time for reflection within the schedule.

To consider how best to optimise simulated surgical learning, guidance can be obtained from theories such as Kolb’s experiential learning theory [21], Vygotsky’s theory of the zone of proximal development [22, 23], and Bandura’s self-efficacy theory [24, 25], among others [26]. In 2005, Kneebone used learning theory to propose four key areas for consideration in relation to procedural skill-based simulation. These were summarised as: sustained deliberate practice in a safe environment consolidated as part of a defined curriculum; accessibility of expert tutors; learning that maps onto real-life experience; and a supportive, motivational, and ‘learner-centred milieu’ [27]. More recently, Stocker et al. have taken a theory-based approach and reported five aspects relating to how simulated team training could be optimised. These have been paraphrased as:

1. Use of key steps of Kolb’s experiential learning: scenario for concrete experience; debriefing with reflection; conceptualisation phase; active experimentation.
2. Scenarios derived from real events, with participants operating at the edge of their comfort zone and challenged to experience failures.
3. Debriefing is fundamental and learners should be facilitated and empowered to engage with critical reflection.
4. Involvement of real team members of different specialties and expertise.
5. Inclusion of the sociocultural context of the team (ideally in-situ simulation) [28].

Combining our findings with learning theory frameworks, there are obvious areas for potential optimisation. Table 5 presents a series of recommendations for optimising LTT based on a combination of simulation best practices and learning theory, supported by empirical evidence specific to LTT. We consider the three most significant recommendations to be: (1) introduction of a brief and debrief to the course schedule; (2) clarifying the learners who have requisite and appropriate need for this type of training, plus restricting attendance only to those with sufficient technical experience; and (3) using LTT only for training in complex procedures where there are no suitable alternative simulator models, with a focus on the animal as a patient and maximisation of contextual and clinical realism.

**Table 5:** Summary of recommendations for optimising simulation training that uses a live animal for surgical training

Simulation Phase	General recommendations	Supporting evidence
Preparing	The preparation phase refers to all activities that take place before a simulated learning event starts, e.g., identifying the learning needs; setting learning objectives; designing the scenarios; choosing simulators, medical equipment; recruiting faculty; writing the schedule; etc. ♦ Define clear learning outcomes or objectives which address a gap in the surgical curriculum.^1^ Surgical learners are attending to try new things and practice rare procedures.^2^ Design scenarios to match these learning outcomes with a focus on integrated technical and non-technical skills.^1^ ♦ Consider whether the choice of simulator matches the educational goal – could another type of simulator be used in place of a live animal to achieve this?^2^ ♦ Consider the learner population with strict(er) criteria for attendance. Surgeons should have sufficient experience to be able to practice more complex skills.^2^ ♦ If multidisciplinary involvement, use real team members in their own role^3^, or appropriate, credible faculty to stand in and augment the team.^4^ ♦ Educators should be experts^2^, with specific training in facilitating learning and creating a supportive and motivational atmosphere.^3,5^ If needed, foster interaction between content experts (clinicians) and process experts (educationalists).^1^	^1^Ker & Bradley, 2010 ^2^*Planning* theme ^3^ Stocker et al., 2014 ^4^ Motola et al., 2013 ^5^ Kneebone, 2005
Briefing	A simulation-specific pre-brief helps to create a safe learning environment. It includes the learning objectives and sets the scene for both learners’ and faculty expectations. ♦ Orientate learners to what is expected in the simulated learning environment with an explicit discussion about what is not similar to reality.^1,2^ This is a key opportunity for learners to understand anatomical differences, environmental differences, etc. ♦ There is an opportunity to discuss the ethics of using animals and rationale for doing so. ♦ Encourage learners and faculty to treat the animal as a patient throughout. Engagement with veterinary staff prior to, and during the simulation, may facilitate this.^3^	^1^ Motola et al., 2013 ^2^ Nestel & Gough, 2018 ^3^*Delivering* theme
Simulating	The simulation activity should have a defined start and end point. ♦ LTT should be learner-centred and not educator-centred.^1^ ♦ Use case studies to create contextual realism^3^, capture clinical variation^4,5^ and map the learning to real-life experiences.^1,2^ ♦ Adapt the scenario to the learner, offering a range of difficulty^4,5^ that challenges the learner without overwhelming them; consider alongside Recommendation 3. ♦ Feedback can be provided in ‘real time’ during the session^4^; however, the focus must be on learner’s needs.^5^ Encouraging observers to make notes to enable specific feedback during debriefing may be valuable to minimise interruptions and maintain learners’ attention within the simulated activity.^6^ ♦ The perception of risk and consequences is important to the experience; failure (i.e., premature death of the animal) should be a possibility in the training process.^3^	^1^ Kneebone, 2005 ^2^ Stocker et al., 2014 ^3^*Experiencing* theme ^4^ Issenberg et al., 2005 ^5^ Motola et al., 2013 ^6^ Nestel & Gough, 2018
Debriefing & Reflecting	Debriefing is a specific form of feedback, which has been found to be the most important part of training using simulation. It is often a collective activity where facilitators address training goals and learning objectives, seek alternative perspectives, and explore learners’ feelings and experiences with the aim of promoting reflection. This is currently absent from LTT. ♦ Instigate debriefing within the LTT program. During debriefing, establish an environment of psychological safety, address key learning objectives, inform learners of reflecting activities and encourage consideration of future learning needs and goals.^1,2,3,4^ ♦ Recording simulations and playing back activity can be helpful to provide feedback and as a debriefing adjunct.^1,4,5^ ♦ Where possible, allow learners to repeat a simulation with active experimentation of learned knowledge^2^, or use deliberate practice techniques to target specific learning goals.^1,6^ ♦ There is minimal benefit to the learners of conducting a post-test assessment; this tests cognitive knowledge rather than change in clinical behaviour or ability.^7^	^1^ Motola et al., 2013 ^2^ Stocker et al., 2014 ^3^ Nestel & Gough, 2018 ^4^ Sawyer et al., 2016 ^5^Ker & Bradley, 2010 ^6^ Issenberg et al., 2005 ^7^*Delivering* theme
Evaluating	This phase refers to the successes and limitations of the simulated learning event, rather than evaluation of the learners. ♦ LTT programs must adapt and change in accordance with learners’ needs.^1,2^ The 3Rs principles should be considered before and after each course to determine whether the minimum number of animals were used, and if their use was appropriately refined.^3^	^1^ Motola et al., 2013 ^2^ Issenberg et al., 2005 ^3^*Delivering* theme

The conceptualisation of LTT as a simulated learning event, which may be a critical change of viewpoint for course directors, clinical educators and learners, means that the key change would be the introduction of a formal briefing and debriefing process to the schedule. Many participants commented that they reflected after the LTT course and on return to clinical practice to a greater or lesser extent, with many only realising the effect years later. By introducing a formalised debrief to the schedule, this individual reflective process is likely to be instigated or hastened. Our participants may have not mentioned debriefing for two reasons: firstly, due to the fact that it doesn’t routinely occur during LTT courses, and secondly, because they are not considering their experience as simulation.

Debriefing is widely thought to be the most critical aspect of simulated learning [29, 30]. It has been stated that “without a post-reflective process, what the participants have learned is left largely to chance, leading to a missed opportunity for further learning, and making the simulation encounter less effective.” [30] Methods of debriefing vary; debriefs can be facilitated by educators or learner-led, structured or semi-structured, occur at different time points in relation to the simulation event and use different conversational techniques [29, 31, 32]. The key characteristic of any debriefing process is an interactive, bidirectional, reflective discussion, which is contrasted with feedback that is typically unidirectional and based on the recipient’s actions [31]. Video-assisted debriefing can profoundly impact participants by grounding the discussion with objective evidence of what happened [29]. However, simply rewatching the activity is insufficient, but must be combined with facilitated discussion [32]. This could open up further opportunities for learning, allowing peers to comment and learn from each other, including capturing missed opportunities to learn from activity that occurs at other operating tables.

If the tenets of the 3Rs are being followed, every opportunity should be made to reduce the number of animals being used for training. If a procedure can be practiced using an alternative simulator model, it should be – animals’ lives must not be needlessly wasted. There is a vast range of alternative options for educators to choose from including, but not limited to: human and animal cadavers, ex-vivo tissues, inanimate mannequins, computer-based and virtual reality simulators and supervised practice on human patients [33, 34]. Our participants considered that fresh frozen human cadavers are beneficial due to the accurate anatomy, with the option for a perfused circulation to simulate bleeding had educational benefits for many. The decision for educators relates to the selection of a suitable tool to achieve the specific objectives of an individual or group of learners within a structured curriculum [34]. 

Our participants believed that learners should be at an appropriate stage of training and with some previous or current experience in managing trauma, to attend. The main technical learning objective is to manage haemorrhage control, with an aim for specific skills (performing a thoracotomy, managing a cardiac injury and repairing major blood vessels). Performing all of these procedures carries the risk that the animal will die prematurely, i.e., before the completion of training. We argue that this in itself is a powerful learning experience, but one at the peripheries of a learners’ comfort zone. Many learners will not have the requisite technical skills or clinical capability to fully engage with this experience, potentially risking their own learning due to cognitive overload, and the animal’s life.

Creative course design could be used to maximise learning while minimising animal use. One option, for example, could be the development of a two-tier course, where early career surgeons observe the surgical team training of more senior colleagues. Senior teams can work under pressure, with the risk of death of the animal. Once their training scenario is completed and they move to debrief, a decision could be made relating to euthanasia. The early career surgeons could use the animal (alive or dead) to focus on technical skills. The use of recently euthanised animals has been demonstrated to be beneficial in simulator-comparison studies [35, 36]. Learners are still able to experience the tissue handling properties and some bleeding, even if the animal is deceased, and will be able to learn from observing senior colleagues. Dedicated expert educators will be able to support their learning in slow-time with a focus on the relevant skills using deliberate practice and providing feedback in the moment as well as debriefing. Meanwhile, the senior team could be undertaking a facilitated video-assisted debriefing session, to reflect on their activities and consider future learning needs.

### Limitations

This study explored learners’ and educators’ experiences of live tissue training for surgical learning in conjunction with simulation theory to consider how LTT could be optimised. The focus is on how training could be optimised for surgeons. There was limited involvement of anaesthetists and emergency physicians within this study and further research is required to explore the benefits of LTT specifically for those learners, and how their learning experience could be optimised. Although the context of this research applies to LTT use in surgical trauma, the recommendations could be extrapolated to other surgical domains and interventional specialties.

This study recruited participants with experiential knowledge of LTT; the small population has a male gender bias and are located within high-income Western countries. The opinions of those who have not, or would not, participate are not reflected within the data. Although the authors exhibit a range of background disciplines and perspectives, none is explicitly against LTT in principle. The findings and associated recommendations are based on the authors’ interpretation of the generated data and our collective knowledge of simulation best practices and literature. Others may nevertheless have alternative interpretations.

Regardless, for justificatory reasons it is important to recognise that efforts must be made to consider educational merit beyond simple skill acquisition if LTT is continued to be used. Assessing simulation learning outcomes, and demonstrating that learning translates to clinical practice is challenging [37]. This study used the knowledge and experience of learners and educators as one possible way to explore and address this, but future research investigating learning outcomes specific to LTT would be of benefit.

## Conclusion

It has been argued that due to the complexity, uncertainty, and emotionally challenging nature of surgery, not all of it can be practiced beforehand [38]. LTT is a type of simulation that, when used intelligently, may be closer to a clinical surgery experience than simulation which uses other, non-living or inanimate models. It is imperative however, that due to inherent ethical concerns associated with using live animals for human benefit, efforts to optimise training should be duly considered.

We have used the experiences of those who have undertaken LTT, combined with learning theories and evidence within the published simulation literature to provide recommendations as to how LTT could be optimised. The most significant recommendations include restructuring the courses to include a brief and a debrief, clarifying the learners who are attending to those that need to address particular and necessary learning objectives, and focusing the learning on the animal as a patient to promote clinical and contextual realism. 

## Supplementary Information

Supplementary Material 1.

## Data Availability

The datasets used and/or analysed during the current study are available from the corresponding author on reasonable request.

## Abbreviations

LTT: Live tissue training
