# Supplementary material for: Optimising the educational utility of live tissue training in trauma surgery
Source: BMC Med Educ. 2025 Sep 10;25:1248. doi: 10.1186/s12909-025-07846-9 (PMC12421745; doi:10.1186/s12909-025-07846-9)
Supplement: Supplementary file 1 — Supplementary Material 1. [file 12909_2025_7846_MOESM1_ESM.docx]

**Introduction**

Thank you for taking the time to participate in this interview. Some background information about the research project was sent to you electronically, and I hope you have had the time to read it.

To facilitate the interview process and data collection, I am recording our conversation today. For your information, only researchers involved with the project will have access to the recordings, they will be stored securely with anonymised file names, and eventually destroyed once they have been transcribed. If you have not already signed the consent form, please do so and return it to me.

I would like to remind you that:

- All information will be confidential;
- Your participation is voluntary and consent to participate can be withdrawn at any time;
- We can pause or stop the interview if you feel uncomfortable;
- We do not intend to inflict any harm via the interview process;
- This interview should last no longer than an hour.

Do you have any questions or concerns before we get started?

**LTT Learner**

You have been invited to speak to me today because you have previously been involved in training delivered using live animals as a simulation model, also known as live tissue training.

My research project is interested in the educational aspects of live tissue training, and this study is interested in perceptions of the training.

Background

In your own words, can you describe your job role?

How long...

- Since you graduated medical school?
- Have you been working in your field?
- Have you been serving in the military? (if applicable)

Question themes

- Learner’s expectations of training and intrinsic/extrinsic motivations
- Learning across different domains (cognitive, psychomotor, affective)
- Impact on interpersonal skills
- Translation of learning to alternative environments
- Ethical implications of training

**LTT Educator**

You have been invited to speak to me today because you have been identified as someone delivers education in the field of trauma management, which involves training delivered using live animals as a simulation model, also known as live tissue training. My research project is interested in the educational aspects of live tissue training, and this study is interested in your perceptions of the training.

Background

In your own words, can you describe your job role?

How long...

- Have you been working in your field?
- Have you been teaching this particular course?
- Have you been serving in the military? (if applicable)

Question themes

- Motivations for teaching on the course
- Specific use and experience of live tissue training on the course
- [Contrast with experience with other simulation modalities]
- Learning across different domains (cognitive, psychomotor, affective)
- Insights on learner expectations
- Ethical implications of training

**Conclusion**

That brings the interview to a close. Thank you for your participation and answering my questions so honestly. I would like to remind you that everything discussed in this interview will remain confidential.

On completion, I will send you some information about future contact from the researchers. This interview will be transcribed, and you can decide whether you would like to receive a copy of it. There will be advice on how to get in touch with us if needed and also some signposting resources if you need any further support.

I will now stop the recording.
